# Impact of substrate choice on human osteoclast differentiation and secretome: Implications for targeted therapeutic development

**DOI:** 10.1371/journal.pone.0333180

**Published:** 2025-10-17

**Authors:** Fernanda D’Amélio, Hugo Vigerelli, Rodrigo Pinheiros Araldi, Adriana da Costa Neves, Álvaro Rossan de Brandão Prieto-da-Silva, Daniel Pimenta, Irina Kerkis

**Affiliations:** 1 Laboratory of Genetics, Butantan Institute, São Paulo, Brazil; 2 The Postgraduate Program in Toxinology, Butantan Institute, São Paulo, Brazil; 3 Centre of Excellence in New Target Discovery (CENTD), Butantan Institute, São Paulo, Brazil; 4 BioDecision Analytics Ltda., São Paulo, Brazil; 5 Laboratory of Biochemistry and Biophysics, Butantan Institute, São Paulo, Brazil; Università degli Studi della Campania, ITALY

## Abstract

Osteoclasts (OCs) exhibit substrate-specific molecular adaptations crucial for bone remodeling. We utilized mass spectrometry and functional enrichment analysis to delineate the proteomic profiles of mature polarized OCs cultured on mineralized versus plastic plates. Our findings reveal that mineralized surfaces promote the expression of proteins specialized for bone resorption and matrix interaction, such as lysosomal enzymes and ion transporters. This environment induces a mature and resorptive phenotype in OCs, enriched in pathways like VEGF/VEGFR signaling and various cytokine pathways. Conversely, OCs on plastic plates display a more diverse proteomic profile, highlighting adaptations in adhesion, proliferation, and stress response pathways, suggesting a focus on cellular maintenance rather than active resorption. Key therapeutic targets for osteoclastogenesis include components of the Hedgehog (Hh) pathway—SHH, DHH, and IHH—with Smoothened (SMO) integral to Hh signaling in OC differentiation. Additionally, Guanine Nucleotide Exchange Factors (GEFs), significantly enriched on plastic plates, are crucial for adapting to non-mineralized environments. Other notable targets include molecular regulators such as NCOR2, which modulates gene expression; NOS1, involved in nitric oxide production and OC function; and XIAP, which influences cell survival. Chromatin remodeling proteins like TACC2 and signaling pathways involving IRS1, MSX1, and AKT are also highlighted. The targets identified in this study are specific to polarized OCs and may not apply to non-polarized OCs or other cell types. These findings underscore the complexity of OC differentiation and function, enhancing our understanding of substrate-specific adaptations and suggesting new strategies for modulating bone metabolism and addressing bone-related disorders.

## Introduction

Osteoclasts (OCs) are multinucleated cells derived from the monocyte/macrophage lineage, crucial for bone remodeling through the resorption of mineralized bone matrix. These cells release enzymes such as tartrate-resistant acid phosphatase (TRAP) and cathepsin K, which degrade the bone matrix and release calcium into the bloodstream, influencing overall bone metabolism [1,2,3]. The differentiation and activity of OCs are markedly influenced by the substrate on which they develop, affecting cell adhesion, signaling pathways, and the surrounding microenvironment.

*In vitro* studies using human peripheral blood mononuclear cells (hPBMCs) to differentiate OCs are crucial for understanding the underlying mechanisms of bone diseases and for developing therapeutic interventions [4]. The principal stages of human OC differentiation *in vitro* involve the initial isolation of hPBMCs, followed by their differentiation into pre-osteoclasts in the presence of macrophage colony-stimulating factor (M-CSF) for approximately 3–5 days. Subsequent exposure to receptor activator of nuclear factor kappa-Β ligand (RANKL) drives pre-osteoclasts’ maturation into fully functional OCs over an additional 5–7 days [5]. These mature OCs are characterized by their multinucleation, expression of TRAP, and the ability to resorb mineralized bone matrix, typically completing differentiation in 10–14 days [6,7]. Functional assessments of OCs include pit assays, where OCs cultured on mineralized plates are evaluated for their ability to resorb bone matrix. This assay quantifies the resorbed area on the mineralized substrate, directly measuring OC activity and bone resorption capacity [3].

Comparative studies of OCs cultured on plastic versus mineralized plates reveal significant differences. OCs on mineralized plates exhibit characteristics that closely mimic physiological conditions of bone remodeling [8], while OCs on plastic plates show greater adaptability to diverse environmental cues. Understanding these substrate-dependent variations is essential for accurately representing OC biology and developing targeted therapies for inflammatory bone diseases [9,10].

In this study, we investigated the impact of substrate choice on the proteomic profile of mature polarized osteoclasts (OCs) by comparing the secretome profiles of OCs cultured on plastic versus mineralized plates. Using advanced mass spectrometry and functional enrichment analysis, we characterized the distinct proteomic landscapes of these polarized OCs. By focusing specifically on the secretomes of polarized OCs, our analysis provides detailed insights into how substrate-dependent adaptations influence their functional plasticity and regulatory mechanisms in bone metabolism. These findings contribute valuable knowledge that could inform the development of innovative therapeutic strategies for bone disorders.

## Materials and methods

### Human PBMCs and OC differentiation protocol

All procedures were performed in compliance with Plataforma Brasil regulations and were approved by the Ethics Committee of the Butantan Institute (CEP protocol No. 1,806,596; approved on November 5, 2016).

PBMCs were isolated using the Ficoll–Paque density gradient centrifugation method (density 1.077 g/mL—Sigma-Aldrich®, USA). Blood samples (20 mL) were collected from healthy male volunteers aged 25–40 years via venipuncture at the cubital fossa (Plataforma Brasil/CEP 1,806,596), then diluted 1:1 with saline (0.9%) and layered onto Ficoll–Paque at a 1:3 ratio in conical tubes. After centrifugation at 400 X g for 20 minutes without acceleration, the PBMCs were carefully isolated. Following two washes with saline, the cells were resuspended in 1 mL of differentiation medium: α-MEM (Thermo Fisher Scientific, Waltham, MA, USA), pH 7.4, supplemented with 10% fetal bovine serum (LGC Biotecnologia, SP, Brazil), 25 ng/mL human M-CSF, 50 ng/mL human RANKL, 5 ng/mL human TGF-β1 (R&D Systems, Minneapolis, MN, USA), and 1 μM dexamethasone (Sigma-Aldrich®, USA).

For osteoclast differentiation assays, 6 × 10^5 PBMCs were plated per 1.9 cm^2 and cultured in 200 µL of differentiation medium. The culture medium was refreshed twice weekly by replacing 50% of the volume for 15 days.

### Tartrate-resistant acid phosphatase (TRAP) Staining

To perform TRAP staining after the differentiation period, the culture medium was first removed, and the cells were gently washed three times with PBS. Subsequently, the cells were fixed by exposure to a solution consisting of 25.5% citrate solution (18 mM citric acid, 9 mM sodium citrate, 12 mM sodium chloride, and surfactant, pH 3.6 ± 0.1), 66.3% acetone, and 2.9% formaldehyde for 30 seconds at room temperature.

The TRAP staining solution, previously prepared according to the manufacturer’s instructions and warmed to 37 °C, was then applied to the fixed cells. After a 1-hour incubation period at 37 °C, the TRAP solution was carefully aspirated, and the cells were washed three times with PBS. Next, the cells were washed three times with preheated (37 °C) deionized water, followed by staining of the nuclei using Gill Hematoxylin Solution No. 3 for 1–2 minutes at room temperature.

To complete the staining process, the cells were rinsed with alkaline water to provide a counter-stain effect. They were included in PBS for analysis. This protocol allows for the visualization and analysis of differentiated cells under light microscopy Nikon Eclipse TS2K-261134, specifically highlighting the presence and distribution of TRAP-positive osteoclasts.

### Pit assay

We employed the culture plates (96-well) coated with an inorganic crystalline calcium phosphate substrate. Each well received 0.2 mL of osteoclast differentiation medium (as described in 4.2.1) and was seeded with 6x105 hPBMCs/well. The cells were incubated in a humidified environment with 5% CO2 at 37ºC. On the 15th day, the resorption activity of OCs was quantified, analyzing the mineralized surface. The medium was aspirated, 100 μL of 10% bleaching solution was added, followed by 15 minutes of incubation at room temperature. The wells were washed three times with distilled water, and then the water was removed completely, allowing the wells to dry for 3–5 hours. Subsequently, the plate was counterstained with the Von Kossa staining kit (Merck© KGaA, Germany) to facilitate visualization of the substrate. The analyses were conducted after capturing images of the entire well with a Nikon stereomicroscope and image analysis software (ImageJ®) to calculate the percentage of reabsorbed surface, enabling the quantification of resorption activity.

### Sample preparation and chromatography

The dried protein samples were reconstituted in 0.1% formic acid (designated as solvent A) to ensure they were in a suitable solution for chromatography. The samples were then injected into a C18 reverse-phase chromatography column (Supelco, 3 μm particle size, 100 Å pore size, and dimensions of 50 mm x 2.1 mm).

A linear gradient elution was performed, starting with 5% solvent B (composed of 90% acetonitrile and 10% water with 0.1% formic acid) and gradually increasing to 40% solvent B over a 66-minute period. The flow rate was maintained at a constant 0.2 mL/min to ensure consistent separation and peak resolution. The eluted components were continuously monitored using a Shimadzu SPD-M20A PDA (photodiode array) detector across the wavelength range of 200–500 nm, allowing for the detection of peptides and proteins based on their UV absorbance.

### Mass spectrometry analysis

For mass spectrometry, an IT-ToF (Ion Trap-Time of Flight) mass spectrometer from Shimadzu (Japan) was employed. The instrument operated in positive ion mode, with an interface voltage set at 4.5 kV, a detector voltage of 1.76 kV, and an interface temperature held at 200°C. Data acquisition covered a mass-to-charge (m/z) ratio range from 50 to 2000, which is suitable for detecting a broad spectrum of peptides and small proteins.

Subsequent MCMS spectra were generated using collision-induced dissociation (CID) with argon as the collision gas. The MSMS mode allowed for fragmentation of selected precursor ions within the same m/z range (50–2000), providing detailed information on peptide sequences and post-translational modifications.

### Protein identification

Protein identification was carried out using PEAKS Studio 7.0 software, which incorporated the InChorus multi-algorithm tool, combining both PEAKS and MASCOT algorithms to enhance identification accuracy. The proteins were matched against public protein databases, including those for Homo sapiens and Squamata species, providing a broad comparison across different species.

### Functional enrichment analysis

Identified proteins were subjected to functional enrichment analysis using the FunRich [11]. This tool allowed for categorization based on molecular function, biological processes, and pathways, providing insight into the potential roles of these proteins in various cellular mechanisms.

### Analysis of possible targets and biological pathways

The data were enriched by the Enrichr software, and twenty-one databases were used to analyze OCs on mineralized plates and twenty-two databases to analyze OCs on plastic plates. The P-Adjusted value, less than 0.005, is taken into account for refinement. After this type of data filtering, graphs were made with the count of pathways and genes that were repeatedly highlighted by the results.

## Results

### Substrate-dependent enrichment of protein localization in osteoclasts: Insights from FunRich analysis

In our OC differentiation assays, hPBMCs were cultured on plastic or mineralized plates in a differentiation medium for 15 days (data not shown) [7]. At the endpoint of differentiation, supernatants were collected and subjected to rigorous mass spectrometry analysis. OCs were characterized using established methods [7], and proteins were identified with precision using mass spectrometry and gas chromatography against a human database (IDPROT).

**Fig 1:**
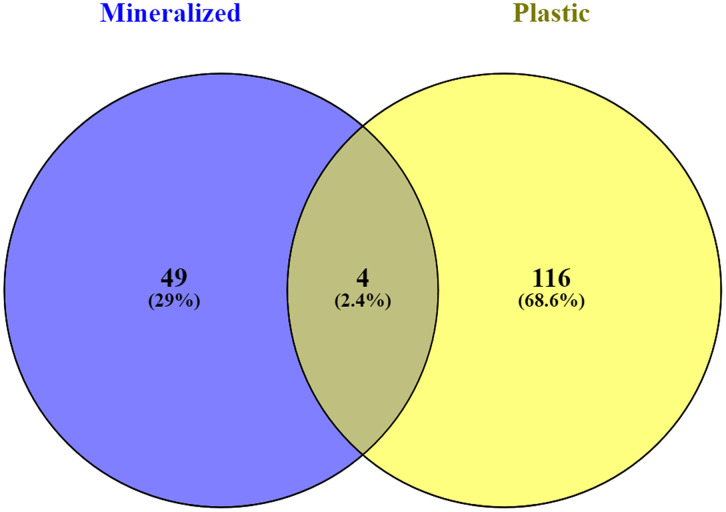
Venn diagram shows osteoclasts: 116 proteins on plastic, 49 on mineralized, with only 4 shared.

Enrichment analysis using FunRich software [11] revealed distinct patterns across biological processes, cellular components, and molecular functions (S1 and S2 Tables). OCs cultured on plastic expressed 116 proteins, whereas those on mineralized surfaces expressed 49 proteins, with only 4 proteins common to both environments (**Fig 1**). Common proteins between the two conditions indicate fundamental OC functions independent of substrate cues. Mineralized substrates appear to direct OCs towards a specialized state optimized for bone remodeling, while plastic substrates elicit a more generalized response (**Fig 1**).

**Fig 2:**
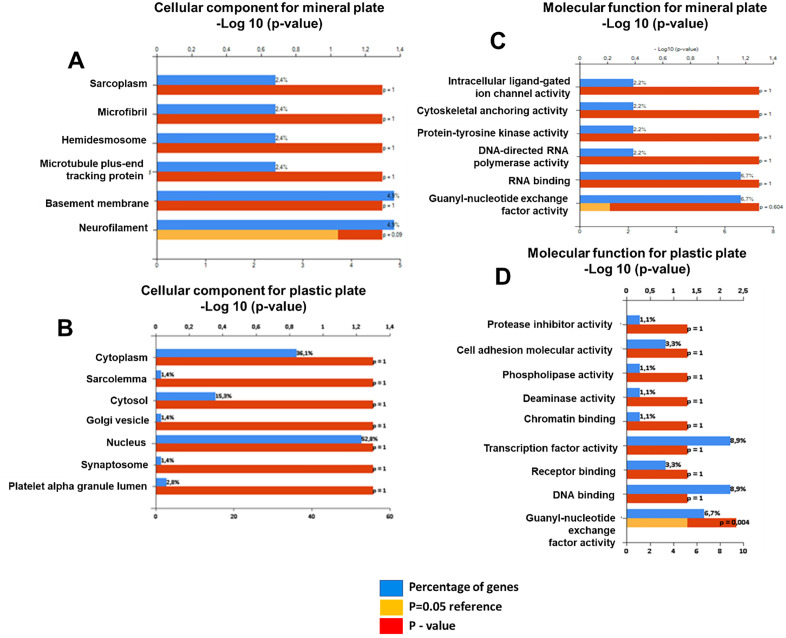
Enrichment analysis of cellular components (A, B) and molecular functions (C, D) of osteoclasts on mineralized versus plastic plates, showing substrate-dependent secretome variations.

Our data provide fascinating insights into the differences in protein expression and localization between OCs differentiated on mineralized versus plastic plates (**Figs 2A and 2B**). The functional similarities between the sarcoplasmic components in OCs and muscle tissue, particularly in calcium ion storage and release, suggest that these OCs may have specialized roles in calcium handling, crucial for their bone-resorbing activities (**Fig 2A**). The presence of microfibrils and crystalline features in the cell membrane is intriguing, as these characteristics are typically associated with plant cell walls and are rarely observed in human cells. This suggests that the mineralized environment may influence the structural organization of OCs, possibly enhancing their interaction with the mineralized matrix. Moreover, identifying hemidesmosomes and proteins related to the basal membrane in OCs differentiated on mineralized plates suggests that these cells may form more robust attachments to their substrate, potentially influencing their resorptive activity and overall function. The similarities in protein functions between the basal membrane and those in OCs may indicate shared roles in cell adhesion and nutrient support. Detecting neurofilament-like structures in these OCs raises interesting questions about potential crosstalk between osteoclasts and the nervous system or the adoption of neuronal features by OCs under certain conditions (**Fig 2A**).

Conversely, OCs differentiated on plastic plates show enrichment of cytoplasmic proteins, with a focus on structures like the sarcolemma, cytosol, and nucleus. The presence of platelet alpha-granules and synaptosomes suggests that these OCs may be more involved in secretory and signaling functions, reflecting a different aspect of OC activity than those on mineralized plates (**Fig 2B**).

While our analysis did not reveal statistically significant differences in protein expression, the observed trends are still meaningful and provide a foundation for future studies. These findings highlight the importance of considering both statistically significant results and emerging patterns when interpreting complex biological data, especially in the context of osteoclast differentiation and function.

### Enrichment analysis of guanine nucleotide exchange factors in osteoclasts differentiated on plastic and mineralized plates

Furthermore, enrichment analysis highlights the significant presence of guanine nucleotide exchange factors (GEFs) (p = 0.004) in the molecular functions of OCs differentiated on plastic but not on mineralized plates (**Figs 2C** **and 3D**). GEFs regulate small GTPases such as Rho and Rac, which are crucial for OC cytoskeletal dynamics, migration, and bone resorption. Dysregulated GEF-mediated signaling could contribute to abnormalities such as osteoporosis or osteopetrosis [12,13].

**Fig 3:**
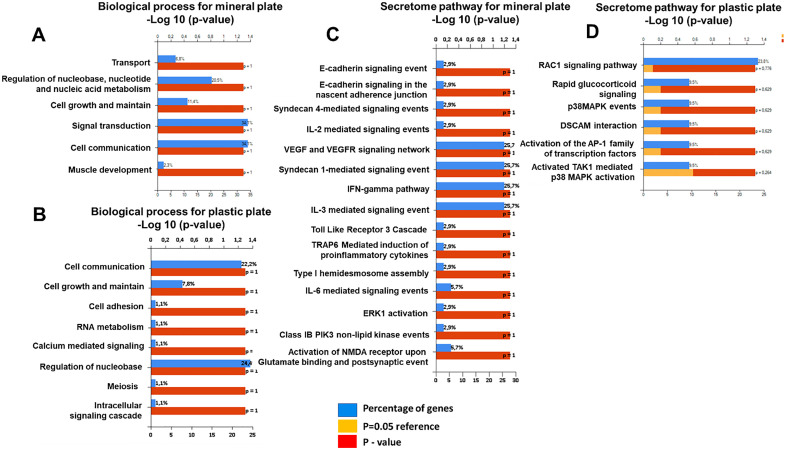
Enrichment analysis of biological processes (A, B) and secretome pathways (C, D) in osteoclasts on mineralized versus plastic plates, showing substrate-dependent variations.

### Adaptations of osteoclasts: Contrasting roles on mineralized and plastic surfaces

Biological process analysis also reveals distinct protein enrichment profiles in OCs differentiated on mineralized versus plastic plates (**Figs 3A, 3B**). OCs on mineralized plates (**Fig 3A**) exhibit significant enrichment in transport processes, emphasizing their role in systemic calcium regulation—a vital function of these cells. These OCs feature an array of membrane transport proteins, including channels, transporters, and pumps, which are crucial for ion exchange and matrix digestion. The maintenance of low pH during bone resorption underscores the importance of these transport proteins for bone differentiation, function, and homeostasis, suggesting they are potential therapeutic targets for bone diseases.

In contrast, OCs on plastic plates show enrichment in RNA metabolism cell communication, growth, adhesion, and RNA metabolism processes, reflecting their adaptation to a less physiologically relevant environment (**Fig 3B**). These cells prioritize calcium-mediated signaling and intracellular cascades, indicating active responses to the artificial substrate through cytoskeletal dynamics and metabolic activity adjustments.

Notably, the enrichment of muscle biology-related processes in OCs on mineralized plates suggests potential crosstalk with muscle tissue development (**Fig 3A**). This connection highlights the integrated nature of bone and muscle tissues, where signaling from OCs during bone remodeling may influence muscle mass, mediated by molecules such as ATP.

The FunRich analysis of proteins secreted by OCs differentiated on mineralized versus plastic plates reveals distinct biological pathways reflective of their substrate-specific adaptations (**Figs 3C**, **3D**). OCs on mineralized plates exhibit enrichment in pathways such as VEGF and VEGFR signaling, syndecan-1 mediated signaling, cytokine pathways (IL-3, IL-6, IFN-gamma), and NMDA receptor activation, highlighting their focus on bone resorption, remodeling, and interaction with the mineralized matrix (**Fig 2D**).

OCs on plastic plates show enrichment in pathways related to cell communication, nucleic acid metabolism, and cellular maintenance, indicative of their adaptation to a non-mineralized environment (**Fig 3D**). These findings underscore how OCs adjust their molecular strategies based on substrate cues, providing insights into bone metabolism and potential targets for therapeutic intervention in bone-related disorders.

**Figs 3A–3D** displays enrichment graphs illustrating differences in biological processes and secretome pathways of OCs differentiated on mineralized versus plastic plates. Secretome data collected on day 14 of differentiation reveal substrate-dependent variations in protein expression and functional profiles, shedding light on how substrate choice influences OC function and secretome composition.

### Integrated databases enrichment analysis reveals substrate-dependent pathways in osteoclasts cultured on mineralized versus plastic plates

Combining 22 databases for plastic plates and 21 for mineralized plates enriches our comparison of OCs cultured on different substrates, leveraging each database’s strengths. Enrichment analyses from CORUM, Elsevier, and Wikipathway highlight critical metabolic and signaling pathways, while Reactome provides detailed insights into molecular interactions. Integrating these analyses identifies consistently enriched pathways and proteins, revealing how OCs adapt to their microenvironment (S2 and S3 Tables).

Based on enrichment analysis of OCs cultured on mineralized plates, several pathways exhibited significant enrichment with adjusted p-values < 0.05 (**Table 1**). These pathways include the Complement Cascade proteins (IGHG3, IGHG4, IGHG1, IGHG2), the PAX9-MSX1 complex (in humans) with MSX1, ITK-SLP-76 complex in anti-TCR stimulated conditions with ITK, and the AMY-1-S-AKAP84-RII-beta complex (in humans) featuring AKAP1, as well as the SLP-76-PLC-gamma-1-ITK complex in alpha-TCR stimulated conditions involving ITK, and finally the Sam68-p85 PI3K-IRS-1-IR Signaling Complex (in humans) (**Table 1**). Enriched protein analysis identifies 10 potential therapeutic targets for bone diseases (**Table 1**): ITK (repeated 2 times), IGHG3, IGHG4, IGHG1, IGHG2, IRS1 (each repeated 1 time), MSX1, and AKT (each repeated 1 time), with statistically adjusted values.

**Table 1:** This table summarizes the pathways and their corresponding proteins that showed significant enrichment in OCs cultured on mineralized plate, based on the adjusted p-values < 0.05 from the analysis.

Pathway description	Database	Adjusted P-value	Gene/ Complex involved
Complement cascade	Bioplanet	0.00360	IGH3, IGHG4, IGHG1, IGHG2
PAX9-MSMX1 Complex (Human)	CORUM	0.00529	MSX1
ITK-SLP-76- Complex, Anti-TCR Stimulated (Human)	CORUM	0.00529	ITK
AMY-1-S-AKAP84-RII-beta Complex (Human)	CORUM	0.00792	AKAP1
SLP-76-PLA-gamma-1-ITK Complex, Alpha-TCR Stimulated (Human)	CORUM	0.00792	ITK
Sam68-p85 PI3K-IRS-1-IR Signaling Complex (Human)	CORUM	0.01055	IRS1

Based on the enrichment analysis of OCs cultured on plastic plates, pathways crucial for osteoclastogenesis, particularly those involving Hedgehog (Hh) signaling, emerge prominently. The Release of Hh-Np (Np – N-terminal Peptide) from secreting cells, Activation of Smoothened (SMO), and various Hh Family Signaling pathways identified across Reactome, Wikipathway, Elsevier, and NCI-nature databases play pivotal roles in regulating these processes. The Nitric Oxide (NO) synthase-dystrophin complex in skeletal muscle, as well as both canonical and non-canonical Hedgehog signaling pathways involving ARRB1/ARRB2, play significant roles in osteogenesis. Additionally, intraflagellar transport mediated by BBSome interactions, the SMRT-SKIP-CBF1 complex, and chromatin remodeling complexes such as TACC2, TACC3, and PCAF are crucial. The Polyadenylation complex also underscores the intricate mechanisms governing mRNA processing and stability in osteoclast development (**Table 2**).

**Table 2:** This table summarizes the pathways and their corresponding proteins and genes that showed significant enrichment in OCs cultured on plastic plate, based on the adjusted p-values < 0.05 from the analysis.

Pathway description	Database	Adjusted P-value	Gene/ Complex involved
Release of Hh-Np from secreting Cell	Reactome	0.00115	SHH
Activation of SMO	Reactome	0.01073	SHH
Hedgehog Signaling Pathway (WP47)	Wikipathway	0.01181	SHH
ARRB1/ARRB/2 non-Canonical signaling and Hedgehog Family	Elsevier	0.00704	SHH
Hedgehog Family - > ARRB1/ARRB2 Canonical Signaling	Elsevier	0.00880	SHH
Hedgehog Family Signaling	Elsevier	0.02608	SHH
Signaling events mediated by the Hedgehog family (Homo sapiens)	NCI-nature	0.00836	SHH
Release of Hh-Np from secreting Cell	Reactome	0.00115	R-HSA-5362798
Activation of SMO	Reactome	0.01073	R-HSA-5635838
Hedgehog Signaling Pathway	Wikipathway	0.01181	WP47
ARRB1/ARRB/2 non-Canonical signaling and Hedgehog Family	Elsevier	0.00704	–
Intraflagelar Transport: BBSome Interaction	Elsevier	0.03001	–
Signaling events mediated by the Hedgehog family (Homo sapiens)	NCI-nature	0.00836	d3a49cee-6195-11e5-8ac5–06603eb7f303
Nitric oxide synthase-dystrophin complex, skeletal muscle (mouse)	CORUM	0.0313	NOS1
ARRB1/ARRB/2 non-Canonical signaling and Hedgehog Family	Elsevier	0.00704	ARRB1/ARRB2
Hedgehog Family - > ARRB1/ARRB2 Canonical Signaling	Elsevier	0.00880	ARRB1/ARRB2
Intraflagelar Transport: BBSome Interaction	Elsevier	0.0300	Bbsome
Signaling events mediated by the Hedgehog family (Homo sapiens)	NCI-nature	0.00836	Hedgehog family
SMRT-SKIP-CBF1 complex (human)	CORUM	0.0313	SMRT-SKIP-CBF1
Chromatin remodeling complex (TACC2, TACC3, PCAF) (human)	CORUM	0.0313	TACC, TACC3, PCAF
Polyadenylation complex (CSTF1, CTF2, CSTF3, SYMPK CPSF1, CPSF2, CPSF3) (human)	CORUM	0.0448	CSTF1, CSTF2, CSTF3, SYMPK CPSF1, CPSF2, CPSF3

**Fig 4:**
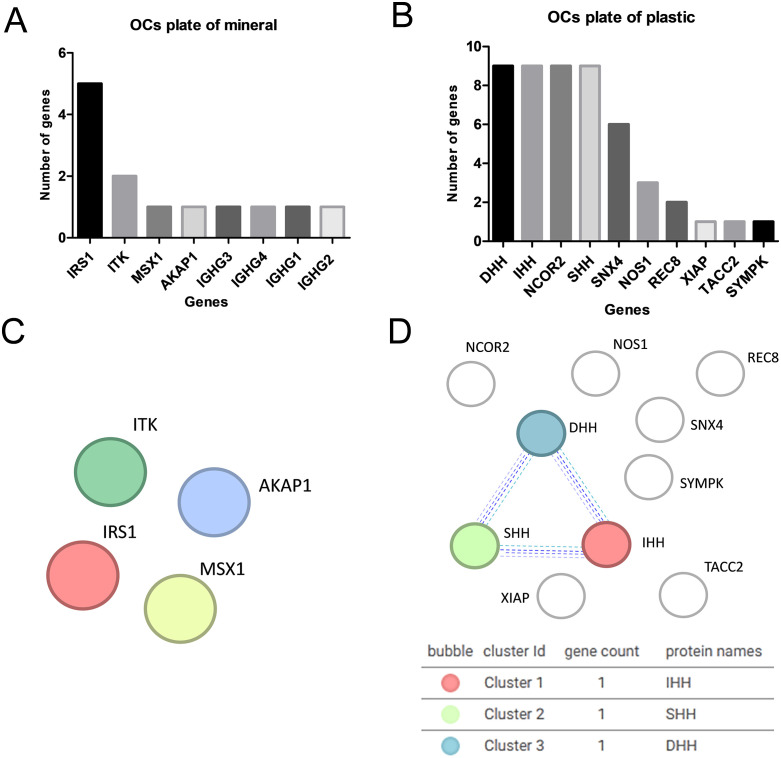
Target definitions for osteoclasts on mineralized (A, C) versus plastic (B, D) plates. STRING analysis shows no clusters on mineralized plates **(C)**, while DHH, IHH, and SHH form a discrete cluster on plastic plates **(D)**.

Enriched gene analysis (on plastic plate) identifies 10 potential therapeutic targets for bone diseases (Fig 4B): DHH, IHH, NCOR2, SHH (repeated 9 times), SNX4 (repeated 6 times), NOS1 (repeated 3 times), REC8 (repeated 2 times), SYMPK, XIAP, and TACC2 (each repeated 1 time), with statistically adjusted values (**Table 2**).

STRING network analysis further supported these findings: in osteoclasts cultured on mineralized plates (**Fig 4C**), the identified proteins did not form interconnected clusters, whereas in osteoclasts on plastic plates (**Fig 4D**), DHH, IHH, and SHH formed a discrete cluster that remained isolated from the other proteins, indicating substrate-specific regulation of the hedgehog signaling pathway in osteoclasts cultured on plastic.

## Discussion

Using polarized OCs provides a more accurate representation of the proteins involved in bone resorption and the regulatory mechanisms active in a mature osteoclast state. This results in a secretome that reflects their enhanced functional capabilities and substrate-dependent adaptations, offering deeper insights into their role in bone metabolism and potential therapeutic targets.

The functional enrichment analysis of proteins secreted by OCs differentiated on mineralized versus plastic plates reveals distinct biological pathways reflective of their substrate-specific adaptations. OCs on mineralized plates exhibit enrichment in pathways crucial for bone resorption, remodeling, and interaction with the mineralized matrix, including VEGF and VEGFR signaling, syndecan-1-mediated signaling, cytokine pathways (IL-3, IL-6, IFN-gamma), and NMDA receptor activation [14–19]. These molecular characteristics suggest that OCs on mineralized plates mature and polarize toward active resorption activities within the bone matrix.

In contrast, OCs differentiated on plastic plates show enrichment in pathways related to cell communication, nucleic acid metabolism, and cellular maintenance. This pattern indicates a focus on maintaining cellular functions rather than active resorption, suggesting that OCs on plastic plates may not be as mature or polarized toward resorptive activities as those on mineralized plates. These results underscore how OCs adjust their molecular strategies based on substrate cues, providing insights into bone metabolism and potential targets for therapeutic intervention in bone-related disorders.

Enrichment analysis reveals that GEFs are involved in osteoclastogenesis on mineralized and plastic plates. Although GEFs were not significantly enriched on mineralized plates, their presence suggests a potential role in bone remodeling and resorption. GEFs were significantly enriched on plastic plates, indicating their importance in adapting cellular signaling to a non-mineralized environment. This highlights GEFs as key players in osteoclast biology and potential targets for future therapies in bone disorders despite the current lack of direct GEF-targeting treatments [20–22].

Enriched protein analysis of OCs cultured on plastic surfaces identifies several potential therapeutic targets for bone diseases, including DHH, IHH, NCOR2, SHH, SNX4, NOS1, REC8, SYMPK, XIAP, and TACC2. These proteins are linked to pathways critical for bone health and regulation. Specifically, DHH, IHH, and SHH, forming a distinct cluster, indicate their pivotal roles in signaling cascades essential for osteoclastogenesis and bone remodeling [23–26]. The Hedgehog (Hh) pathway is critical in skeletal development, guiding OC differentiation and bone formation from embryonic stages to adulthood [23,24]. SHH is particularly notable for its therapeutic potential and has been extensively studied in diseases such as osteosarcoma and skeletal dysplasia [26]. While studied for its role in embryonic skeletal patterning, DHH remains less explored as a therapeutic target than SHH. IHH, similarly implicated in bone biology and development, has also received less attention in therapeutic research [25]. Continued investigation into DHH and IHH may reveal their full potential for therapeutic interventions in bone diseases and related conditions. Activation of Smoothened (SMO) and various Hh family signaling pathways are integral for mediating essential cellular responses fundamental to OC development and function, highlighting their potential as therapeutic targets for modulating bone metabolism and related diseases [27–29].

Beyond Hh signaling, other pathways implicated in osteoclastogenesis include a diverse array of molecular complexes and regulatory processes [30,31]. This encompasses the Nitric Oxide (NO) synthase-dystrophin complex in skeletal muscle, both canonical and non-canonical Hedgehog signaling pathways involving ARRB1/ARRB2, intraflagellar transport mediated by BBSome interactions, and specific Hh family signaling events [32–34]. Additionally, the SMRT-SKIP-CBF1 complex and chromatin remodeling complexes (TACC2, TACC3, and PCAF) significantly contribute to gene regulation and cellular differentiation processes critical for OC development [35,36]. The involvement of the polyadenylation complex further underscores the intricate mechanisms governing mRNA processing and stability in osteoclastogenesis [37].

The NCOR2 protein, also known as Nuclear Receptor Corepressor 2, plays a significant role in osteoclastogenesis by modulating gene expression in response to various signals, thereby influencing the differentiation and activity of OCs in bone remodeling processes [38–40]. NOS1, which regulates nitric oxide production, impacts bone remodeling and OC function [41–43]. Among less frequently noted proteins, XIAP stands out for its anti-apoptotic functions and regulation of cell survival pathways, influencing OC survival and function [44–47].

Enriched protein analysis of OCs cultured on mineralized surfaces highlights IRS1 as a potential therapeutic target for bone diseases, reflecting its involvement in bone metabolism and skeletal health through insulin signaling pathways [48–50]. Conversely, ITK’s role in bone diseases remains less explored despite its primary function in T-cell signaling and immune responses. Emerging research suggests ITK may influence OC differentiation, which is critical in bone remodeling and associated with autoimmune and inflammatory conditions [51,52]. IGHG3, IGHG4, IGHG1, and IGHG2 encode different immunoglobulin heavy chain gamma (IgG) subtypes, typically associated with immune responses rather than direct bone disease involvement. MSX1, involved in craniofacial and skeletal development, and AKT, critical for cell survival and growth, are relevant to bone biology and may hold therapeutic potential for bone diseases [53–56].

## Conclusions

^‌‌^

^‌‌^

In conclusion, our analysis highlights distinct molecular adaptations of mature, polarized OCs based on substrate type. OCs on mineralized plates are enriched in pathways critical for bone resorption and remodeling, such as VEGF/VEGFR signaling and cytokine pathways, indicating a mature, resorptive phenotype. Conversely, OCs on plastic plates show enrichment in pathways related to cellular maintenance, reflecting a focus on sustaining cellular functions rather than active resorption.

Key therapeutic targets for mature OCs include components of the Hedgehog (Hh) pathway (e.g., SHH, DHH, IHH, and Smoothened) and GEFs, which are crucial for adaptation to non-mineralized environments. Additionally, regulators like NCOR2, NOS1, and XIAP, along with chromatin remodeling proteins such as TACC2, and signaling pathways involving IRS1, MSX1, and AKT, offer potential therapeutic avenues. These findings underscore the complexity of OC differentiation and function and suggest new strategies for modulating bone metabolism and addressing bone disorders.

The targets identified are specific to polarized OCs and may not apply to non-polarized OCs or other cell types. This specificity enhances our understanding of substrate-specific adaptations in OC biology and highlights potential therapeutic targets for bone-related conditions.

## Supporting information

S1 Table Complete table with the data analyzed using enrichment analysis. https://maayanlab.cloud/Enrichr/
(XLSX)

S2 Table Enrichment analysis of the pathways and their corresponding proteins and genes in osteoclasts cultured on plastic plates combining 22 databases. Reactome, Bioplanet, Wikipathway, KEEG, ARCHS4, Elsevier, MsigDB, Biocarta, Human, NCI-Nature, Bioplex, HUmap, PPIHub, KEA 2015, Virus Host, NURSA, CORUM, HMS LINCS, SUBcell, The Kinase Library, PFOCR and Rummagene.
(XLSX)

S3 Table Enrichment analysis of the pathways and their corresponding proteins and genes in osteoclasts cultured on mineralized plates combining 21 databases. Reactome, Bioplanet, Wikpathway, ARCHS4, Elsevier, Biocarta, NCI-Nature, Bioplex, huMAP, PPIhub, KEA, Kinase perturbations down, Kinase perturbations up, Virus Host, CORUM, HMS LINCS, DEPOD, Glygen, The Kinase Library, PFOCR and Rummagene.
(XLSX)
